# After surgery: the effects of life-saving caesarean sections in Burkina Faso

**DOI:** 10.1186/s12884-015-0778-7

**Published:** 2015-12-23

**Authors:** Véronique Filippi, Rasmané Ganaba, Clara Calvert, Susan F. Murray, Katerini T. Storeng

**Affiliations:** London School of Hygiene and Tropical Medicine, London, UK; AfricSanté, Bobo-Dioulasso, Burkina Faso; King’s College London, London, UK; University of Oslo, Oslo, Norway

**Keywords:** Caesarean section, Burkina Faso, Africa, Sequelae, Maternal morbidity, Obstetric complications, Near-miss, Postpartum care, Costs

## Abstract

**Background:**

In African countries, caesarean sections are usually performed to save mothers and babies’ lives, sometimes in extremis and at considerable costs. Little is known about the health and lives of women once discharged after such surgery. We investigated the long-term effects of life-saving caesarean section on health, economic and social outcomes in Burkina Faso.

**Methods:**

We conducted a 4 year prospective cohort study of women and their babies using mixed methods. The quantitative sample was selected in seven hospitals and included 950 women: 100 women with a caesarean section associated with near-miss complication (life-saving caesareans); 173 women with a vaginal birth associated with near-miss complication; and 677 women with uncomplicated vaginal childbirth. Structured interviews were conducted at 3 months, 6 months, 12 months and 3 and 4 years postpartum. These were supplemented by medical record data on delivery and physical examinations at 6 and 12 months postpartum. The lives and experiences of 21 women were documented ethnographically. Data were analysed with multivariable logistic regressions, using survival analysis and thematic analysis.

**Results:**

The physical effects of life-saving caesareans appeared to be similar to women who had an uncomplicated childbirth, although 55 % of women with life-saving caesareans had another caesarean in their next pregnancy. The negative effects were generally economic, social and reproductive when compared to vaginal births, including increased debts (AOR = 3.91 (1.46–10.48) and sexual violence (AOR = 4.71 (1.04–21.3)) and lower fertility (AOR = 0.44 (0.24–0.80)) 4 years after life-saving caesareans. In the short and medium term, women with life-saving caesareans appeared to suffer increased psychological distress compared to uncomplicated births. They were more likely to use contraceptives (AOR = 5.95 (1.53–23.06); 3 months). Mortality of the index child was increased in both near-miss groups, independent of delivery mode. Ethnographic data suggest that these consequences are significant for Burkinabe women, whose well-being and social standing are mostly determined by their fertility, marriage strength and family links.

**Conclusions:**

Life-saving caesareans have broad consequences beyond clinical sequelae. The recent policy to subsidise emergency obstetric care costs implemented in Burkina Faso should help avoid the majority of catastrophic costs, shown to be problematic for women undergoing emergency caesarean section.

## Background

Caesarean sections are an essential component of emergency obstetric care for preventing maternal and newborn deaths. In high-income settings, caesareans sections are easily accessible; they can be planned if necessary, and are generally considered as “safe”. However, in low-income settings, caesarean sections are often carried out in emergencies and in sub-optimal quality of care circumstances. In Africa and most parts of South East Asia, population level caesarean section rates remain below the minimum threshold of 5 % suggested in international monitoring guidelines to avoid deaths and severe morbidity in the mother [1]. Whereas unnecessary surgical procedures in upper and middle class African women are increasingly common [2], access to emergency, life saving caesarean sections remains inadequate among the poorest women and in rural areas.

Long distances to facilities, lack of human resources, inadequate supplies and equipment, costly procedures and fear of surgical delivery have been put forth as explanations for these low levels of caesarean section. Cultural factors may also play a role. In Benin, for example, prolonged and difficult labour is a sign that a woman has been unfaithful to her husband, and can result in “treatments” being sought outside the formal health sector [3]. In low income settings, healthcare users perceive a direct causal link between the caesarean sections and maternal deaths [3–5]. Studies from different African settings have found that many women fear an emergency caesarean section because it is expensive and potentially devalues women in their two principal roles: productive and reproductive [6]. In addition, some women see caesarean section as an unnatural way to give birth or as a personal failure [5].

While caesarean sections in Africa are most often performed to save women’s lives or prevent serious complications, there are risks associated with the procedures. In the short term, both emergency and planned caesarean sections may be associated with infections and adverse outcomes in the mothers including increased mortality and psychological distress [6–11]. Caesarean sections are also associated with increased risks of placenta praevia in the next pregnancy [12] and in some contexts with reduced fertility, whether voluntary or acquired [13]. In some situations caesarean sections protect against short term urinary incontinence [14] and prolapse at older ages [15], but it is not clear the extent to which this protective effect might occur when women have been in labour for a long time before the caesarean. Negative pyscho-social consequences on self-esteem, emotional responses and mother-infant interactions have been documented, linked both to cultural norms as well as women’s perceived degree of control over the delivery process [16].

While there has been a long scientific debate on the relative long term benefits and sequelae of vaginal birth and caesarean section in high and upper middle income settings [11, 17], few studies have documented the outcomes of these procedures in low income settings [18]. In this paper, we examine the long-term impact of a life-saving emergency caesarean section on social, economic and health outcomes in Burkina Faso, West Africa.

## Methods

This article draws on a larger, inter-disciplinary longitudinal study that seeks to understand the long-term (3 to 4 years) impact of severe obstetric complications and the costs of treating such complications on the health, economic and social well-being of women. The study was conducted in Burkina Faso, one of the most impoverished countries in the world. Women in Burkina Faso had high maternal mortality (300 per 100,000 live births), one of the highest fertility rates in the world (total fertility rate above 6) and under-five mortality of 129 per 1000 live births in 2010 [19, 20]). Burkina Faso’s district health system functions poorly, including for the delivery of emergency obstetric care. The national caesarean section rate was below 1 % in 2002–2006 and 1.8 % in 2007–2011, suggesting a low level of elective caesarean section at the population level [20]. The government started to implement a policy to subsidise the cost and reduce user fees for emergency obstetric care and skilled birth attendance in 2006, during the time of our study. The impact of this policy on the cost of care for women has been substantial although women’s households pay fees that are much higher than those set by the policy [21].

Our study design combined a prospective epidemiological cohort study (2004–2010) with a nested longitudinal ethnographic study, comparing the experiences of women with near-miss complications to those with uncomplicated childbirth [22–25]. Near-miss complications are severe acute events, such as haemorrhagic or septic shock, which carry a very high risk of death for women, unless hospital care is provided [26]. We recruited a total of 1014 women from seven hospitals in six towns across Burkina Faso between November 2004 and March 2005 and, after obtaining the women’s informed consent, extracted data from their medical records. We chose to recruit the sample from health facilities to minimise ascertainment bias for the near-miss exposure, as women’s reports of severe morbidity in the community can be inaccurate [27]. Women were eligible if they lived within a 30 km radius of the facility; this radius was selected to facilitate follow-up of the women. We applied diagnostic-based operational definitions of near-miss events for five categories of complications (anaemia, hypertension, haemorrhage, dystocia, infections, and others) using clinical signs, symptoms or management criteria which could be found retrospectively in medical records and which were agreed locally during a workshop with obstetricians and epidemiologists on the basis of a literature review. For haemorrhage, for example, women were classified as near-miss if they had abnormal internal or external blood loss accompanied by shock, blood transfusion, hysterectomy or coagulation problems. Full details of the operational definitions are available on request [28]. Data on whether the women delivered vaginally or by caesarean section were also obtained from medical records. Uncomplicated childbirth was defined as a vaginal delivery in a health facility, without complications during pregnancy and before discharge, and ending in a live birth at term. A woman with uncomplicated childbirth may, however, have had an episiotomy, forceps or vacuum extraction.

### Quantitative data collection and analysis

The women were administered structured questionnaires by trained interviewers at home within a week of discharge, and again at 3, 6 and 12 months postpartum and also in the third year and fourth year after the end of pregnancy. Medical information on weight, anaemia and hypertension were obtained during a physical and gynaecological examination at 6 months and 12 months post-pregnancy, and through simple medical tests conducted by the interviewers at home in year 3 and 4 (anaemia (hemocue), blood pressure, body mass index (BMI)). Individuals were classified as overweight if their BMI was greater than 25, and anaemic if their hemocue haemoglobin result was less than 7 g/dl. Hypertension was defined as a blood pressure reading greater than 140/90 mmHg. Data on urinary incontinence were also gathered at the 6 and 12 months medical examination, and on prolapse at 12 the month examination by the gynaecologists. At all time points, self-reported data were obtained on difficulties in doing routine tasks (e.g. preparing meals or going to the market) in the past month, and psychological distress was assessed using the K10, a scale to screen for poor mental health as well as other indicators [29]. Women also reported whether the index child was still alive at the time of the interview. In this study, mortality of the index child refers to deaths in the community after hospital discharge.

During these interviews self-reported data were also gathered on social outcomes, including domestic violence and marital disharmony, and economic outcomes such as unpaid debts. The frequency of divorce was also examined using women’s self-reports and data on reasons for loss to follow-up recorded in our database. Data were also collected on whether women had resumed sexual activity (at the 3, 6 and 12 month interviews), whether women used contraceptives (at all time points) and on the outcome of any subsequent pregnancies (at 3 and 4 years).

All statistical analyses were conducted in Stata 13. After excluding 64 women with early pregnancy loss who were in our original near-miss sample, we compared long term health, social and economic outcomes for three groups of women in order to separate the influence of the delivery procedures from the influence of the severity of the complications: 1) women who experienced a near-miss complication and who delivered by caesarean section (“life saving caesareans”) (100 women); 2) women who experienced a near-miss complication and who delivered vaginally (173 women); and 3) women with a healthy pregnancy and an uncomplicated delivery (677 women including one woman who had a forceps delivery). Loss to follow-up by the 4th year was 30 % overall, with the highest loss to follow-up in the caesarean section group (35.0 %) (usually because of migration outside the study area or the respondents’ death [23]). Chi-square tests were used to test for differences between the groups with respect to the baseline study characteristics, such as age and parity. A survival analysis was conducted to compare the rate of infant and child survival after hospital discharge between the study groups. For all other outcomes, logistic regression was performed to calculate odds ratios comparing the life saving caesareans group vs. the uncomplicated childbirth only. Adjusted odds ratios were produced controlling for the influence of parity and wealth quintiles, the latter of which was derived from a score based on each woman’s household asset ownership.

### Qualitative data collection and analysis

Qualitative data derive from open-ended, in-depth interviews with 21 women sub-sampled from the epidemiological cohort. This sub-sample included a systematic sample of all the women in the life-saving caesarean group from three regional hospitals serving a rural and peri-urban populations (*N* = 20), plus a randomly generated sample (*N* = 8) of the women recruited from a tertiary-level hospital. We were unable to interview seven of these women: three had moved residence and could not be located; one could not be interviewed due to language barriers and; three were now deceased. The remaining 21 women were interviewed 3–4 years after the caesarean section by M. Akoum in Dioula, in the local language or in French.

The interviews were audio-recorded and transcribed, with simultaneous translation to French. KS, SM and MA each read the interview transcript and conducted initial thematic analysis, which they refined during a 2-week residential analysis workshop. The analysis is also informed by a broader ethnographic study conducted with a sub-sample of 82 of the women in the epidemiological cohort [6, 30]. Five of the 21 women had participated in this broader study, and had thus participated in up to three follow-up in-depth interviews in the first year after their caesarean section.

### Ethical clearance

Three ethical committees approved the study: Centre Muraz (initial 12 months study) and the national “Comité d’Ethique pour la Recherche en Santé” (follow up in year 3 and 4) in Burkina Faso and the London School of Hygiene and Tropical Medicine (both phases) in the UK. Women gave written consent: after reading or listening to the consent procedure, they signed the consent form using handwriting or fingerprint.

## Results

### Baseline characteristics at the time of selection (quantitative sample)

Women’s average age was 25.6 years, and was similar across the three comparison groups (Table 1). A higher proportion of women in the near-miss group with vaginal childbirth were in their first pregnancy. Women with life-saving caesarean were wealthier than other women with a near-miss complication but poorer than women with uncomplicated childbirth (*p* < 0.001). They were more likely than other women with a near-miss complication to have been diagnosed with uterine rupture and bandl ring (a sign of obstructed labour). The proportion of perinatal deaths was 21.0 % amongst women with life-saving caesarean section and 30.6 % amongst women with near-miss and vaginal delivery.

**Table 1:** Distribution of baseline characteristics of the study population stratified by the delivery group

		Uncomplicated (*N* = 677)	Near-miss caesarean (“Life saving caesarean”) (*N* = 100)	Near miss - other (*N* = 176)	*P*-value^g^
Parity^a^	0–1	37.4	46.0	45.1	
	2–3	37.0	32.0	27.2	
	4+	25.6	22.0	27.7	0.09
Gravidity^b^	1	33.6	38.4	38.7	
	2–3	38.9	33.3	30.1	
	4+	27.5	28.3	31.2	0.25
Age group^c^	<20	18.8	20.0	22.1	
	20–24	30.5	28.0	33.1	
	25–29	24.6	23.0	18.6	
	30+	26.1	29.0	26.2	0.71
Wealth quintiles^d^	Most poor	15.7	22.1	30.2	
	Second	18.0	25.3	23.1	
	Third	21.7	21.1	16.0	
	Fourth	22.1	16.8	16.6	
	Least poor	22.6	14.7	14.2	<0.001
Near-misscomplication	Hypertension	NA	30.9	31.3	0.97
	Haemorrhage	NA	23.7	37.3	0.03
	Dystocia	NA	42.3	4.8	<0.001
	Infection	NA	17.5	38.0	<0.001
	Anaemia	NA	13.4	41.0	<0.001
Birth weight^e^	Singleton				
	≥2500 g (normal)	86.7	78.4	53.5	
	<2500 g (low)	13.3	21.6	46.5	<0.001
	Twin				
	≥2500 g (normal)	27.3	14.3	50.0	
	<2500 g (low)	72.7	85.7	50.0	-
Apgar score at 5 minutes^f^	Singleton				
	≥7 (normal)	99.5	82.8	80.0	
	<7 (low)	0.5	17.2	20.0	<0.001
	Twin				
	≥7 (normal)	100	90.0	100	
	<7 (low)	0	10.0	0	-

^a^1 missing values

^b^2 missing values

^c^3 missing values

^d^35 missing values

^e^47 missing values for singleton deliveries; 1 missing value for one twin

^f^125 missing values for singleton deliveries; 1 missing value for one twin

^g^The *p*-value compares the proportions in all three groups with the exception of the medical complications which are only compared in the two near-miss groups

### Health consequences

The proportion of women (excluding those pregnant at the time of the interview) overweight and with hypertension increased significantly across the follow-up period in all three groups (*p*-value = 0.001and 0.02, respectively). This was particularly evident among women with life-saving caesareans, almost half (43 %) of whom were overweight by the fourth year, whereas 9 % had hypertension (Table 2). There was, however, only statistical evidence for an association between life-saving caesarean and risk of hypertension at 3 and 4 years after delivery when compared with women with an uncomplicated birth, adjusting for other risk factors (adjusted odds ratio (3 years): 3.14, 95 % confidence interval (CI):1.06–9.36) (Table 3). We did not detect significant differences between the groups for other physical health outcomes, including prolapse and incontinence, but it is worth noting that prolapse is a frequent diagnosis (overall prevalence of 25.0 %).

**Table 2:** Percentage of women with each complication at each time point, by near miss status

	3 month interview			6 month interview			12 month interview			3 year interview			4 year interview		
	No complication	Near-miss - other	Near-miss - caesarean	No complication	Near-miss - other	Near-miss - caesarean	No complication	Near-miss - other	Near-miss - caesarean	No complication	Near-miss - other	Near-miss - caesarean	No complication	Near-miss - other	Near-miss - caesarean
PHYSICAL HEALTH															
Proportion overweight (BMI >24 kg/m2)^a^	.	.	.	22.6	16.5	19.5	21.9	21.3	23.9	32.1	22.5	35.4	41.9	33.7	43.4
Hypertension (>140/90 mmHg)^a^	.	.	.	2.1	2.9	2.4	1.3	1.7	1.4	3.2	5.9	7.6	3.6	7.1	8.9
Severe anaemia (hemocue haemoglobin <7 g/dl)^a^	.	.	.	0.5	1.6	2.5	0.4	0	2.7	1.6	3.0	1.5	.	.	.
Difficulties doing 1 or more routine task	10.6	16.3	16.7	9.8	15.9	12.5	7.5	10.9	4.8	18.5	19.0	20.0	15.8	23.4	18.3
Urinary incontinence	.	.	.	13.8	14.7	14.5	1.6	3.1	1.3	.	.	.	.	.	.
Prolapse	.	.	.	.	.	.	26.2	20.3	24.3	-	-	-	.	.	.
MENTAL HEALTH															
Depression – % at or above the K10 14 cut-off score	9.6	14.9	18.5	6.6	11.6	7.0	6.7	10.9	6.1	6.4	5.1	8.7	10.4	10.4	11.7
Have you recently thought about taking your life	5.5	10.9	8.6	3.4	6.5	6.8	5.6	11.6	12.0	4.0	5.1	7.1	.	.	.
Feeling average/not good/not good at all today	10.8	15.4	12.9	11.6	17.4	19.3	9.6	14.5	9.6	15.4	23.9	8.6	14.5	19.6	20.0
RELATIONS WITH HUSBAND/PARTNER															
Relationship has gotten worse	12.2	17.5	12.4	12.3	21.8	12.5	12.1	11.4	17.1	15.2	14.4	14.3	13.2	12.6	12.5
Partner humiliated you in front others	5.7	7.6	6.7	10.0	13.7	12.5	8.3	10.6	9.2	12.6	8.7	14.5	10.9	9.5	8.3
Partner forced you to have sexual relations when you did not want to	2.7	2.1	0	3.5	1.6	0	2.5	4.1	2.6	2.5	0	1.8	1.2	3.2	6.3
Divorce	.	.	.	.	.	.	.	.	.	.	.	.	9.2	12.5	15.7
ECONOMIC															
Still have not paid off hospital costs from delivery	1.5	4.7	8.0	1.3	3.7	10.5	3.7	10.7	15.1	1.4	2.7	8.6	.	.	.
FERTILITY															
Reported contraceptive use^b^	55.6	46	83.3	58.3	49.3	64	51.5	42.3	65.5	39.5	34.4	37.7	57.5	41.3	60.4
Resumed sexual intercourse	28.3	35.5	25.8	58.3	58.3	56.8	73.8	69.4	72.6	.	.	.	.	.	.
Subsequent birth	.	.	.	.	.	.	.	.	.	43.6	51.4	30.4	52.7	58.8	40.0
Subsequent live birth	.	.	.	.	.	.	.	.	.	43.6	51.4	29.0	52.1	57.8	38.3

^a^Women who report being currently pregnant at each time point are not included in calculations of anaemia, hypertension and being overweight

^b^Denominator is number of women who were sexually active at months 3,6 and 12 and all women at year 3 and year 4 and were not pregnant

**Table 3:** Odds ratios comparing each outcome in the near-miss caesarean group to the uncomplicated delivery

	3 month interview		6 month interview		12 month interview		3 year interview		4 year interview	
	Crude OR	Adjusted OR^3^	Crude OR	Adjusted OR^c^	Crude OR	Adjusted OR^c^	Crude OR	Adjusted OR^c^	Crude OR	Adjusted OR^c^
PHYSICAL HEALTH										
Proportion overweight (BMI >24 kg/m2)^a^	-	-	0.83 (0.46–1.51)	1.22 (0.64–2.34)	1.12 (0.61–2.04)	1.58 (0.81–3.09)	1.16 (0.67–2.00)	1.56 (0.86–2.84)	1.06 (0.60–1.89)	1.20 (0.64–2.25)
Hypertension (>140/90 mmHg)^a^	-	-	1.16 (0.26–5.29)	1.51 (0.31–7.30)	1.05 (0.13–8.70)	1.38 (0.15–12.73)	2.49 (0.87–7.08)	3.14 (1.06–9.36)	2.63 (0.92–7.48)	3.23 (1.10–9.54)
Severe anaemia (haemocue haemoglobin <7 g/dl)^a^	-	-	4.84 (0.80–29.41)	NA	7.56 (1.05–54.54)	6.39 (0.82–50.02)	0.92 (0.11–7.61)	0.85 (0.10–7.16)	-	-
Difficulties doing 1 or more routine task	1.69 (0.92–3.10)	1.80 (0.97–3.34)	1.32 (0.67–2.62)	1.22 (0.60–2.51)	0.62 (0.22–1.77)	0.59 (0.21–1.70)	1.10 (0.59–2.06)	1.07 (0.55–2.05)	1.20 (0.60–2.40)	1.11 (0.53–2.32)
Urinary incontinence	-	-	1.06 (0.55–2.04)	1.20 (0.61–2.36)	0.81 (0.10–6.48)	0.75 (0.09–6.05)	-	-	-	-
Prolapse	-	-	-	-	0.91 (0.52–1.60)	0.96 (0.52–1.75)	-	-	-	-
MENTAL HEALTH										
Depression – % at or above the K10 14 cut-off score	2.13 (1.18–3.83)	1.84 (0.98–3.43)	1.06 (0.43–2.56)	1.01 (0.41–2.50)	0.91 (0.35–2.37)	0.89 (0.34–2.36)	1.39 (0.56–3.45)	1.57 (0.62–3.95)	1.13 (0.49–2.63)	0.86 (0.34–2.15)
Have you recently thought about taking your life	1.63 (0.73–3.63)	1.63 (0.73–3.68)	2.05 (0.81–5.20)	1.33 (0.44–4.01)	2.31 (1.10–4.85)	2.44 (1.14–5.20)	1.87 (0.68–5.12)	2.19 (0.77–6.23)	.	.
Feeling average/not good/not good at all today	1.23 (0.64–2.36)	1.17 (0.59–2.33)	1.82 (1.02–3.27)	2.00 (1.08–3.67)	1.00 (0.46–2.18)	1.02 (0.47–2.24)	0.51 (0.22–1.22)	0.41 (0.16–1.06)	1.47 (0.74–2.91)	1.33 (0.65–2.72)
RELATIONS WITH HUSBAND/PARTNER										
Relationship has gotten worse	1.01 (0.51–1.98)	0.91 (0.45–1.85)	1.02 (0.50–2.06)	1.00 (0.49–2.04)	1.50 (0.78–2.86)	1.59 (0.82–3.08)	0.93 (0.42–2.04)	0.87 (0.37–2.03)	0.94 (0.38–2.31)	0.94 (0.38–2.33)
Partner humiliated you in front others	1.21 (0.49–2.95)	1.13 (0.42–3.00)	1.28 (0.63–2.62)	1.36 (0.66–2.80)	1.12 (0.49–2.58)	1.22 (0.52–2.85)	1.18 (0.53–2.62)	1.28 (0.57–2.88)	0.74 (0.26–2.17)	0.75 (0.25–2.19)
Partner forced you to have sexual relations when you did not want to	-	-	-	-	1.07 (0.24–4.79)	1.11 (0.24–5.06)	0.72 (0.09–5.61)	0.78 (0.10–6.35)	5.69 (1.32–24.61)	4.71 (1.04–21.30)
Divorce	-	-	-	-	-	-	-	-	1.85 (0.98–3.48)	1.75 (0.91–3.37)
ECONOMIC										
Still have not paid off hospital costs from delivery	5.57 (2.06–15.02)	4.82 (1.73–13.41)	9.16 (3.43–24.44)	7.03 (2.50–19.79)	4.60 (2.13–9.93)	4.13 (1.86–9.17)	4.56 (1.73–12.02)	3.91 (1.46–10.48)	-	-
FERTILITY										
Reported contraceptive use^b^	4.00 (1.31–12.17)	5.95 (1.53–23.06)	1.27 (0.69–2.35)	1.46 (0.72–2.93)	1.79 (1.01–3.17)	2.25 (1.19–4.26)	0.93 (0.53–1.61)	0.99 (0.56–1.75)	1.13 (0.61–2.08)	1.34 (0.69–2.61)
Resumed sexual intercourse	0.88 (0.54–1.44)	0.92 (0.54–1.56)	0.94 (0.61–1.45)	1.05 (0.66–1.66)	0.94 (0.58–1.53)	1.06 (0.64–1.76)	-	-	-	-
Subsequent birth	-	-	-	-	-	-	0.53 (0.31–0.92)	0.40 (0.22–0.72)	0.57 (0.33–0.99)	0.44 (0.24–0.80)
Subsequent live birth	-	-	-	-	-	-	0.57 (0.33–0.97)	0.43 (0.24–0.78)	0.60 (0.35–1.03)	0.47 (0.26–0.84)

^a^Women who report being currently pregnant at each time point are not included in calculations of anaemia, hypertension and being overweight

^b^Denominator is number of women who were sexually active at months 3,6 and 12 and all women at year 3 and year 4 and were not pregnant

^c^Adjusted for parity and wealth quintile

Women who had undergone life-saving caesarean section were more likely to present signs of psychological distress at 3 months postpartum, and were significantly more likely to report suicidal thoughts at the 12 months interview compared with women with an uncomplicated delivery. Suicidal thoughts were more common among women with near-misses (whether caesarean section or vaginal delivery) at all time points. These adverse mental health outcomes relate to the social and economic consequences of near-misses, which are pronounced for women who have experienced life-saving caesarean sections as they explained in the in-depth interviews. Women described caesarean sections as a particularly frightening and traumatic experience with an unknown cause or remedy; an illness that a woman could not treat herself but for which she has to place her trust in health staff.

**Fig. 1:**
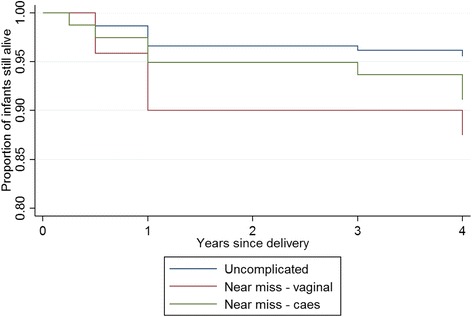
Survival of infants in the 4 years post-delivery by maternal near miss status

High child mortality may contribute to the negative psychological outcomes associated with near miss experiences. The babies of women with near-miss (both vaginal and caesarean delivery) have higher post-discharge child mortality than the babies of women with uncomplicated delivery (Fig. 1). Over the 4 year period, the rate ratios for deaths were 2.29 (95 % CI: 1.01–5.21; *p*-value = 0.04) for babies in the near-miss caesarean group compared to uncomplicated vaginal birth group; and 3.13 (95 % CI: 1.68–5.81; *p*-value < 0.001) for babies in the near-miss vaginal delivery group compared to uncomplicated vaginal birth group (Fig. 1).

### Social and economic consequences

Life-saving caesarean sections are associated with immediate catastrophic healthcare expenditure and long-lasting effects on impoverishment and debt. Despite the difficulty of measuring self-reported hospital costs, we have previously shown that the cost for a life-saving caesarean section was 63,000CFA (approximately £64.88) compared with 11,724CFA for an uncomplicated delivery in hospital and 29,179CFA for delivery with a near-miss (without a caesarean section) [30]. Our follow-up analysis shows that women with life-saving caesarean section more frequently reported debts related to the delivery at all time points, up to 4 years after the procedure (Table 3), when close to one in 10 of these women had not finished paying their debts related to the caesarean.

A life-saving caesarean not only has the potential to impoverish the household, but can also compromise women’s economic independence. Women often had minor personal savings that they used to pay for the emergency procedure or follow-up care. Many were active in informal trade before the delivery, but had been forced to sell stock to meet the costs of care, making it difficult to resume their productive activities in the aftermath. Following costly caesareans, a woman sometimes took personal responsibility for household costs previously paid for by their husbands, such as children’s school fees or medicines, to “compensate” for the husband’s outlay on her care or to mitigate household perceptions that she was an economic burden.

The stigmatising nature of being labelled an economic burden was hard to dispel, not just because of the direct costs incurred at the time of the delivery, but because women who have had a caesarean section were told that they will need to give birth in hospital in the future, will need medical follow-up, and will need to rest, all elements likely to reduce income and contribution to the household economy. In so far as men invest in pregnancy a woman’s caesarean section can mean that it no longer becomes economic to do so.

At the same time, the impact of catastrophic expenditure associated with life-saving caesareans does not necessarily deter future pregnancies. One woman, for instance, incurred catastrophic costs in the first caesarean section that caused long-lasting family tensions and shame, but still went ahead with a new pregnancy because of the social importance of becoming a mother. Some women saved up for the costs of the subsequent pregnancy, but most left it to the father of their child (as is customary). However, men also found it difficult to prepare for such costs, and often waited until the event before mobilising resources by borrowing money or selling animals or other assets.

Following life-saving caesareans women often feared – and sometimes experienced – relationship breakdown and the stigmatising position of being considered “dépensière - costly or wasteful. In some cases this resulted in divorce or separation, or in men taking a new wife or sexual partner. Indeed, the quantitative data show that both sexual violence (but not other forms of violence) and to a lesser extent divorce were more common among women with near-miss and caesarean section in year 4. In Burkina Faso, many women still practice abstinence after childbirth, and this could explain the delayed effect of sexual violence.

Women who had undergone life-saving caesareans can struggle to find a new partner following the breakdown of a relationship, not least because of the likely need for costly medical treatment in a subsequent pregnancy. In-depth interviews revealed that the extent to which a woman’s history of caesarean section affected her chances of establishing a new relationship and getting married depended on the man’s situation – how many children he already had and whether he was looking for someone to look after them, whether he wanted more children, and whether the new relationship was based on love.

Despite the difficulties, some women did establish new relationships, such as one teenager who had become pregnant following rape and who then survived an obstetric complication thanks to an expensive emergency caesarean section. Desperate to no longer be a burden on her aging parents, she agreed to live with an older man in her neighbourhood who was looking for a young second wife. When her situation in his household deteriorated, she left him and returned to her parental home, before eventually meeting a young man with whom she got pregnant and settled happily. She went on to have an uncomplicated second delivery and a healthy child.

### Subsequent fertility and childbearing

According to the in-depth interviews, women in Burkina Faso fear an emergency caesarean section not only because it is expensive and potentially dangerous, but also because it can compromise future childbearing. Women who underwent life-saving caesarean sections were told that they can only have a maximum of two or three caesarean sections in their lifetime. Health workers often advised such women to “rest” from pregnancy either by taking contraceptives or abstaining from sex. This helps explain why they used contraception more frequently than the other women at all time points in the study (crude and adjusted odd ratios significantly higher at the 3 and 12-month interviews).

At the same time, during in-depth interviews, women often spoke about their own desire to get pregnant again soon to “prove” their continued fertility and to secure their position within the household. Some went against the medical advice to delay pregnancy, proceeding with a subsequent pregnancy quickly despite the known risks and probable high costs.

Half of the women in the qualitative sub-sample had a repeat pregnancy. All of these women had antenatal care and all were advised to go to a large health centre or hospital to deliver. They were also advised to go to a bigger facility in third trimester for an ultrasound. Nearly all went to hospital to deliver in their subsequent pregnancy. They described the hospital as a place to be saved (but also as a place to be swindled, claiming that lower level health staff steal maternity ward patients’ money).

A successful pregnancy and delivery next time around was perceived by these women as a way to mitigate many of the social problems raised by the near-miss and caesarean section. By contrast, they also faced the risk that further problems in the subsequent pregnancy might entrench their reduced social standing. One woman, for instance, suffered ill-health during her new pregnancy and miscarried in the fifth month, acquiring the label of a woman ‘at risk’ and now feeling that she was “condemned” to not have children. Another woman, whose emergency caesarean section ended with a stillborn child had got pregnant again immediately. When she developed problems and required medical care again during this new pregnancy, her husband accused her of having got pregnant with another man. He saw her health problems as confirmation of the “doubts” he had about his wife, and even accused her of having had a caesarean section “in order to waste his money”.

Our quantitative analysis provides evidence (p < 0.05) that women who had a life saving caesarean were less likely to have a subsequent birth compared with women with uncomplicated childbirth. For example, 38.3 % of women with a life saving caesarean section reported having had a subsequent live birth 4 years later, compared with 57.8 % of other women with a near-miss complication, and 52.1 % of women who had an uncomplicated delivery. Unsurprisingly, of those that had a subsequent pregnancy, 55.6 % of women in the life saving caesarean group reported having had another caesarean, much higher than in either the other near-miss or uncomplicated group (10.2 and 5.4 %, respectively).

Women who were unable or unwilling to have another child after caesarean section adopted a range of social survival strategies. Some stayed in an unhappy marriage for financial security, while others worked intensely to ensure existing children’s education to secure their own future, or set out to earn independent income to protect themselves against loss of financial contributions from male partners.

### Subsidy policy

The economic – and social - consequences described above were no doubt mitigated in subsequent pregnancies of women who had experienced a near-miss complication by the recent introduction of the government subsidy of the cost of emergency obstetric care including caesarean section. Despite a low level of general awareness of this policy, those who underwent repeated caesarean sections remarked on it being much more affordable than previously. One woman, for instance, claimed she paid “three times less” for the second caesarean section. At the same time, at the time of our study, there were considerable implementation challenges related to the subsidy policy. For instance, women claimed that health workers over-prescribe and sometimes steal small amounts of drugs and medical items which they resell to patients, thereby undermining the policy goal of increased financial access to routine and emergency obstetric care.

## Discussion

Caesarean sections saved the lives of many women in our study. While the physical trauma of caesarean sections appears to be short-lived (as it was not captured at survey 3 months postpartum) or similar to the physical impact of uncomplicated childbirth, near miss caesareans appear to entail a range of striking consequences for women. These include higher risk of debts, sexual violence, reduced or “sub-fertility” and divorce 4 years after the event. In the short and medium term, there are indications that women suffered psychological distress. They were also more likely to use contraceptives (as recommended by their health providers [31]) to delay or avoid further births [24].

The fact that a woman who has had a caesarean section may be considered to have become a financial drain could be one of the reasons why there is a higher proportion of contraceptive use and abstinence in the caesarean group in the initial period of follow-up as shown in a previous article [24]. Both women and men also appear to have understood from their behavior and in depth interview that women are at an increased risk of a subsequent caesarean section for medical indications. While the 2006 reduction of user fees in Burkina Faso has reduced the financial burden experienced by household [21], women with caesareans are still paying an average of 18,160CFA, and the financial and social consequences described are likely to persist.

Our study measured fertility by documenting the proportion of women with subsequent birth. The lower fertility experienced by women after a caesarean section has been shown elsewhere and attributed to the psychological and/or physical trauma of childbirth, including rational decision making and wound infections [13, 32]. A systematic review and meta-analysis estimated a 10 % increase in sub-fertility (measured by time intervals) among women with caesarean compared to vaginal delivery [32]. Another systematic review found an 11 % lower birth rate [13], an effect which is smaller than the decrease in birth rate found in our study, possibly because our study includes particularly traumatic caesarean sections as women had near-misses. Many (55 %) of the women with life saving caesarean section who become pregnant again had another caesarean section within the study period. This is lower than in the high income settings such as the UK (around 63 %) and USA (90 %), but similar to the proportion observed in research studies included in a Cochrane review (57 %) and therefore implies a standard of obstetric care which is neither too cautious nor too interventionist [33, 34]. The lack of expected protective effects of caesarean section against prolapse [10] might be related to the high prevalence of the condition in this high fertility population and to the fact that many caesarean sections were done in emergency after the women had been in labour for some time.

The babies of women with a near-miss complication, whether or not associated with caesarean, are also more likely to have high mortality post-discharge. These disturbing child mortality results in both near-miss groups are partly explained by the high proportion of low birth weight and low apgar score in newborns. Low apgar score may be due in part to distress because of late intervention which in turn relates to poor access to care. The particularly high mortality among children of women with near-miss who did not have caesareans may reflect that they were poorer and were less able to access healthcare in a timely manner. In other words, these women may have experienced the so-called first and second delays, in deciding to seek care and in reaching hospital-level care, respectively [35]. Previous results from our cohort study show an increased mortality in women with near-miss complication up to 4 years after the event related to chronic ill health and failures of the health systems to cater for their complicated social and healthcare needs [23, 36]. Combined with the findings of this paper, this suggests the need for more intense, longer, and more holistic postnatal primary health care for women with near-miss complications and their babies.

Our study findings must be interpreted in relation to the strengths and limitations of our methods. The main strengths are the multi-disciplinary nature of the study and the range of outcomes documented. However, there was relatively high loss to follow up by 4 years, with some evidence to suggest that those loss to follow up were younger and had fewer children than those who stayed in the study (data not shown). Furthermore, the small number of caesarean sections meant it was not possible to control for all identified confounders, and for some outcomes (for example divorce) we may not have had sufficient power, potentially leading us to incorrectly conclude that there is no association. The small numbers of women in certain categories of our sample may also explain the variations in trends for some of the outcomes, such as debts. Whilst near miss women are representative of their population (if the hypothesis that they would be very likely to die if they did not receive hospital care is correct), women with uncomplicated childbirth are not representative of the general population of women with normal childbirth. This is because they chose to deliver in a referral hospital, while many women in Burkina Faso still deliver at home and the majority of facility births are in health centres. In addition, it would have been useful to further contrast the outcomes of interest in samples of women with planned caesarean section to understand the effects related to the procedure as opposed to the emergency.

## Conclusion

The long-term adverse consequences of life saving caesarean sections in Burkina Faso are primarily economic and social. These economic and social consequences are significant for Burkinabe women, whose well-being and social standing are, to a large extent, determined by the strength of their marriage and family links. The recent policies to subsidise the costs of emergency obstetric care implemented in several African countries including Burkina Faso are likely to reduce these consequences, by reducing delays and avoiding the majority of catastrophic costs. However the battle against maternal mortality and severe obstetric complications has not yet been won in these African countries, where the life-time risk for maternal deaths remains 1 in 42, compared to 1 in 2900 in Europe. This suggests that many women who experience a near-miss complication will continue to have emergency caesarean sections which are very costly for themselves, both in financial and social terms.
